# Parsnp 2.0: scalable core-genome alignment for massive microbial datasets

**DOI:** 10.1093/bioinformatics/btae311

**Published:** 2024-05-09

**Authors:** Bryce Kille, Michael G Nute, Victor Huang, Eddie Kim, Adam M Phillippy, Todd J Treangen

**Affiliations:** Department of Computer Science, Rice University, Houston, TX 77005, United States; Department of Computer Science, Rice University, Houston, TX 77005, United States; Department of Computer Science, Rice University, Houston, TX 77005, United States; Department of Computer Science, Rice University, Houston, TX 77005, United States; Genome Informatics Section, Center for Genomics and Data Science Research, National Human Genome Research Institute, National Institutes of Health, Bethesda, MD 20892, United States; Department of Computer Science, Rice University, Houston, TX 77005, United States; Department of Bioengineering, Rice University, Houston, TX 77030, United States

## Abstract

**Motivation:**

Since 2016, the number of microbial species with available reference genomes in NCBI has more than tripled. Multiple genome alignment, the process of identifying nucleotides across multiple genomes which share a common ancestor, is used as the input to numerous downstream comparative analysis methods. Parsnp is one of the few multiple genome alignment methods able to scale to the current era of genomic data; however, there has been no major release since its initial release in 2014.

**Results:**

To address this gap, we developed Parsnp v2, which significantly improves on its original release. Parsnp v2 provides users with more control over executions of the program, allowing Parsnp to be better tailored for different use-cases. We introduce a partitioning option to Parsnp, which allows the input to be broken up into multiple parallel alignment processes which are then combined into a final alignment. The partitioning option can reduce memory usage by over 4× and reduce runtime by over 2×, all while maintaining a precise core-genome alignment. The partitioning workflow is also less susceptible to complications caused by assembly artifacts and minor variation, as alignment anchors only need to be conserved within their partition and not across the entire input set. We highlight the performance on datasets involving thousands of bacterial and viral genomes.

**Availability and implementation:**

Parsnp v2 is available at https://github.com/marbl/parsnp.

## 1 Introduction

As a result of the democratization of sequencing technology, the quality and quantity of publicly available assembled genomes has grown at an unprecedented rate (Kille *et al.* 2022, Nurk *et al.* 2022). Multiple genome alignment, the process of identifying nucleotides which share a common ancestor across a set of genomes, is a critical step in typical comparative genomics workflows. However, tools that aim to compute multiple genome alignments have struggled to keep up with the ever-increasing rate of growth of available data.

To reduce the complexity and maximize the signal of comparative genomics analysis for a group of closely related genomes, it is often helpful to reduce them to their highly conserved orthologous regions. By nature of being highly conserved and vertically inherited, orthologous regions are well suited for constructing phylogenies as well as identifying core functions and regulatory regions within microbial genomes.

While core-genome analysis has been enabled by many tools over the last decade, Parsnp (Treangen *et al.* 2014) is one of the few capable of identifying and reporting the core genome and all of its variation from the sequences alone. Alternative methods, such as Roary (Page *et al.* 2015) and Read2Tree (Dylus *et al.* 2023), rely on genome annotation and annotated databases to map to orthologs and identify core-genome SNPs. Importantly, Parsnp constructs the core genome through multiple sequence alignment as opposed to pairwise alignment, therefore avoiding any reference bias.

To identify the core-regions of a set of genomes, Parsnp first identifies maximal unique matches (MUMs), sequences which are identical across all genomes and unique within each genome, to anchor the global core-genome alignment. Parsnp then identifies syntenic regions, also known as locally collinear blocks (LCBs), based on these MUMs, performs a recursive MUM search, and then passes each syntenic region on to multiple sequence alignment to obtain the SNPs for each genome.

Parsnp has been widely adopted since its release in 2014. Despite this, there have been no major releases for nearly a decade, leading to a consistent growth in open issues and feature-requests ranging from easier installation to new post-processing options. In addition, the recent influx of massive intra-species assemblies has identified a core issue in the Parsnp method: the length and quantity of MUMs often decreases as the number of input sequences grows due to minor variation and assembly artifacts. As a result, the identified core-regions in massive datasets, particularly those of low-quality or high diversity, are more fragmented and less abundant.

In a new release of Parsnp, we address a significant number of open issues and feature requests, fix subtle but impactful bugs, and provide a new divide-and-conquer workflow which allows Parsnp to identify core-regions which were not captured due to the aforementioned MUM dropout on massive datasets. In addition, our divide-and-conquer approach is trivially parallel and requires substantially less memory than the original Parsnp workflow.

## 2 Materials and methods

### 2.1 Partitioning workflow

As a core region in Parsnp is defined as a region that is present in all input sequences, any core region is also present in all sequences for any subset of the input. Parsnp v2 offers a partitioning workflow which takes advantage of this by partitioning the input genomes up into groups of equal size and running Parsnp v2 on each group, using the same reference for each partition. Then, Parsnp v2 reports the common regions across all partitioned core genomes as the final core genome.

The advantage of this workflow is that it is much more parallel and requires significantly less memory. In addition, it can lead to larger core genomes for very large input datasets since very large datasets typically suffer from MUM dropout, as mentioned previously.

### 2.2 Identifying final core-genome coordinates from partitions

In order to identify the coordinates of the final core genome with respect to a reference sequence, we compute the intersection of the aligned reference regions for each partition. Let q=n/p be the number of partitions of *n* query genomes with *p* genomes per partition and $R_{i}=\{(b_{1},e_{1}),(b_{2},e_{2}),\ldots \}$ be the begin and end coordinates of the reference for each of the LCBs in partition *i*.

We compute the interval intersection of all *q* interval sets to obtain the coordinates of the final core-genome intervals, $R^{*}$. The output alignments of each partition are then trimmed to match the coordinates of $R^{*}$. At the end of this step, each partition will have an alignment file with $\left|R^{*}\right|$ LCBs, where each LCB corresponds to one of the intervals in $R^{*}$.

The trimmed output alignments of each partition are then stitched into a combined alignment of all input sequences, using the fact that the alignment columns represent an equivalence relationship between the reference and query sequences for each position. Special care must be taken for insertions with respect to the reference, though. To handle this particular case, we align inserted sequences across partitions using SPOA (Vaser *et al.* 2017). More details on this step can be found in the Supplementary.

### 2.3 Additional Parsnp v2 features

In addition to the aforementioned partitioning workflow and bug-fixes, there are many new improvements which greatly improve Parsnp’s utility. Until 2018, Parsnp was not available on the Bioconda channel and required a manual build of a custom MUSCLE library (Edgar 2004) in addition to the Parsnp binary. This, with the additional requirement of Python 2 and other dependencies not listed on Bioconda, made for a laborious and nontrivial installation process. In order to ameliorate these issues, we updated Parsnp to support Python 3, added Parsnp as well as its dependencies to Bioconda, and in effect rid the need for users to obtain and build Parsnp and its multiple dependencies.

We have also added numerous additional options to Parsnp’s interface which dictate the input, output, and internal pipeline of the Parsnp method. With the exception of the partitioning option, the workflow of Parsnp remains the same as in the original version. A figure detailing the steps of Parsnp can be seen in Supplementary Fig. S1. Users can now provide their query inputs through regular expression CLI matching and text files, output VCF files directly, use FastANI (Jain *et al.* 2018) for genome recruitment, use FastTree2 (Price *et al.* 2010) for phylogeny reconstruction or skip the step as a whole, and more. These improvements greatly increase the utility of Parsnp v2, making it suitable for more custom end-user analysis.

## 3 Results

### 3.1 Validation using simulated genomes

We used ALF (Dalquen *et al.* 2012) to simulate 500 genomes at varying rates of divergence. All parameters were set to the “MSA” preset with the exception that all genome-level events, duplication, loss, inversion, translocation, horizontal gene transfer, and rate variability, were turned on and set to their default values. The RTL distance parameter was set to 0.03, 0.2, and 0.3, resulting in datasets with average divergences of roughly 1%, 3%, and 6%, respectively. Parsnp was run once without partitioning and also once with a partition size of 50. CoreDetector (Fruzangohar *et al.* 2023), a recent core-genome alignment method, was also run for comparison, setting the expected divergence level to the ground-truth average divergence.

To provide an objective validation of Parsnp’s ability to capture evolutionary signal compared to CoreDetector, we ran RAxML (Stamatakis 2014) on the output alignments of each method for each dataset and compared the resulting species trees to the corresponding ground-truth species tree provided by ALF. We used the ETE 3 Toolkit (Huerta-Cepas *et al.* 2016) to obtain the normalized Robinson-Foulds distance for each tree. The value of the normalized Robinson-Foulds (nRF) distance represents the number of bipartitions which differ between the two trees normalized by the total number of internal branches across both trees. The results of this experiment are presented in Table 1.

**Table 1.** Normalized Robinson-Foulds distance between predicted and ground-truth phylogenies across different levels of divergence.^a^

	Divergence		
	1%	3%	6%
Parsnp (p=50)	0.00	0.01	0.02
Parsnp (p=n)	<0.01	0.05	0.07
CoreDetector	0.02	0.05	0.20

a ALF (Dalquen *et al.* 2012) was used to simulate three sets of 500 genomes each at different levels of divergence. Parsnp and CoreDetector were run on each dataset and the core-genome alignment from each tool was passed to RAxML to predict a species tree. Normalized Robinson-Foulds distances were then calculated using ETE 3 (Huerta-Cepas *et al*. 2016).

Parsnp with partition sizes of 50 (p=50) yielded a perfect tree when the genomes diverged by only 1% on average, and nonpartitioned Parsnp (p=n) produced a nearly perfect tree (nRF ¡0.01). CoreDetector’s predicted tree had a small distance of 0.02. However, Parsnp (p=50) was much more robust to increasing levels of divergence than the other two methods. Notably, when the divergence was 6%, the Parsnp (p=50) yielded 3x fewer incorrect branches than the Parsnp (p=n) and 10x fewer incorrect branches than CoreDetector. The size of the core-genome produced by Parsnp (p=50) was comparable to CoreDetector’s, however CoreDetector consistently produced around half as many LCBs.

### 3.2 Benchmarking on real data

**Figure 1.**
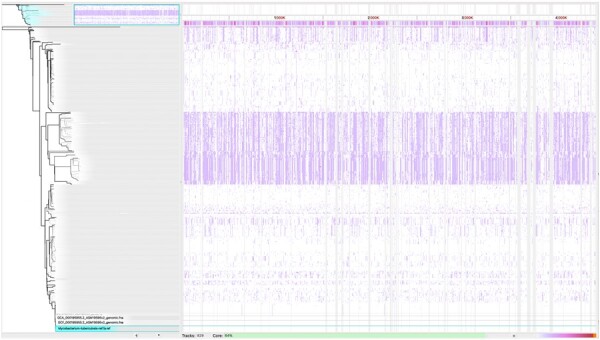
Gingr visualization of a Parsnp (p=250) alignment on 828 *M.tuberculosis* genomes. When zoomed out, the visualization displays a heatmap of single-nucleotide variants. Base-level resolution views of the alignments become visible when inspecting smaller regions.

All available complete assemblies for *Staphylococcus aureus*, *Mycobacterium tuberculosis*, *Klebsiella pneumoniae* were downloaded from NCBI. This resulted in groups of 2729, 828, and 4326 assemblies, respectively. In addition, we obtained a random sample of 100 000 complete SARS-CoV-2 genomes from GenBank, each containing at most 10 ambiguous nucleotides. For each group of assemblies, Parsnp (commit 1d3fbcc) was run on a server with 128 GB of memory and 8 processors with partition sizes *p* of 50, 100, and 250 genomes as well as without partitioning (p=n). CoreDetector was also run for comparison. As Parsnp has the convenience of producing .ggr files which can be viewed with Gingr, we demonstrate a visualization of the *M.tuberculosis* core genome in Fig. 1.

### 3.3 Performance and core-genome characteristics

For each run, the peak memory usage as well as wall-clock and CPU time are reported in Table 2. In addition to the runtime performance, Table 2 also reports the weighted average divergence from the reference across all LCBs, where each LCB is weighted by the number of nucleotides it contains, the size of the core genome, and the number of LCBs.

**Table 2.** Performance of Parsnp on pangenomes of different sizes compared to CoreDetector.^a^

Dataset	Method	Wall time (m)	CPU time (m)	Memory (Gb)	Weighted average reference divergence (%)	Average core length (kbp)	LCB count
*M.tuberculosis* n=828 3.5 Gbp	Parsnp (p=50)	32	214	**21.0**	0.03	1727	4976
	Parsnp (p=100)	38	253	46.3	0.03	2368	5995
	Parsnp (p=250)	62	297	42.3	0.03	2776	5815
	Parsnp (p=n)	60	262	46.0	0.03	2997	4873
	CoreDetector	**25**	**71.3**	26.7	0.07 (0.21)	2669 (3231)	1212 (1323)
*S.aureus* n=2729 7.4 Gbp	Parsnp (p=50)	**69**	488	**13.9**	0.47	830	2725
	Parsnp (p=100)	76	521	23.9	0.46	785	2421
	Parsnp (p=250)	110	626	68.5	0.43	694	2064
	Parsnp (p=n)	181	857	96.9	0.38	414	1414
	CoreDetector	**69**	**163**	54.2	0.50 (0.54)	656 (898)	457 (513)
*K.pneumoniae* n=4326 24 Gbp	Parsnp (p=50)	**183**	**1350**	**31.7**	0.28	1314	6207
	Parsnp (p=100)	202	1433	58.9	0.27	1258	5574
	Parsnp (p=250)	298	1749	123.8	0.26	1138	4674
	Parsnp (p=n)	NA	NA	>128	NA	NA	NA
	CoreDetector	NA	NA	>128	NA	NA	NA
SARS-CoV-2 *n*=100 000 3.2 Gbp	Parsnp (p=50)	55	**313**	7.76	0.15	25.7	134
	Parsnp (p=100)	**54**	316	8.57	0.15	25.4	133
	Parsnp (p=250)	85	380	**6.89**	0.15	25.0	123
	Parsnp (p=n)	NA	NA	NA	NA	NA	NA
	CoreDetector	NA	NA	NA	NA	NA	NA

a Parsnp was executed with different partition sizes *p* on each set of genomes. Total number of genomes, *n*, and dataset size shown is also shown. For each dataset, the best wall time, CPU time, and peak memory usage are bolded. Mean reference divergence is defined as 1 minus the weighted average of the average nucleotide identity across all LCBs, where the weight is the total number of nucleotides in the LCB. The average core length is the average number of nucleotides present in the core alignment for each genome, and the LCB count is the number of LCBs reported. The results of CoreDetector before removing LCBs with indels larger than 300 bp are reported in parentheses (Parsnp did not produce any such LCBs). Both the nonpartitioned run of Parsnp and CoreDetector failed on the *K.pneumoniae* due to excessive memory use. For SARS-CoV-2, the nonpartitioned run of Parsnp failed to produce a core genome after 88 min due to lack of MUMs in the anchoring step and CoreDetector halted after 13 h due to a runtime error.

Large indels represent sequences not present in all of the input genomes and therefore are not considered part of the core genome, and as such, Parsnp will not report them. To enable a more appropriate comparison, LCBs with indels >300 nucleotides were removed from CoreDetector’s output. After this adjustment, the size of the core region reported by CoreDetector was comparable to Parsnp, but the average divergence was consistently higher relative to Parsnp i.e. Parsnp was able to produce a better-aligned core genome of roughly the same size.

In line with the observation of large gaps in CoreDetector’s output is the fact that CoreDetector consistently reports fewer LCBs and more aligned bases. While this aggressive merging of smaller LCBs often provides a simpler view of synteny, it may come at the cost of aligning through nonconserved or noncore regions, leading to inaccuracies in downstream analysis. Indeed, while the average LCB size for CoreDetector was nearly twice the average LCB size for Parsnp (p=50) across the simulated datasets, there were far more incorrect branches in the CoreDetector-predicted phylogenies than for Parsnp (p=50) (Table reftable: tree-validate).

Table 2 shows that the memory and runtime advantages of the partitioned approach can be substantial and can enable alignment on hardware that might otherwise have been resource-prohibitive. While the wall-clock time used by Parsnp (p=50) and CoreDetector were comparable in the two datasets where CoreDetector completed successfully, CoreDetector required up to 3x less CPU time than Parsnp (p=50). However, as most modern machines have at least 8 cores, the wall-clock time is more representative of practical settings. For the partitioned mode of Parsnp, the memory usage scales linearly with both the partition size and the number of threads used; users can increase the number of threads used in return for shorter wall-clock times so long as they have sufficient memory available.

In three of the four datasets, smaller partitions result in a larger core genome with very little cost to the alignment quality. The remaining alignment for *M.tuberculosis* suggests that there are genomic datasets where partitioning decreases the core-genome coverage. One distinguishing feature of this dataset is that it contained more LCBs than the other datasets (Table 2) despite having fewer genomes and lower divergence. This has been observed in a prior study on *M.tuberculosis* genome alignment as well (Elghraoui *et al.* 2023).

Nonpartitioned Parsnp (p=n) is likely at an advantage in this scenario; higher LCB counts due to high levels of rearrangements (and other factors) result in an increase in the number of synteny breakpoints. Nonpartitioned Parsnp has a global view across all input genomes and is able to both filter out potential spurious MUMs and perform a recursive MUM search across all genomes to help mitigate the effect of high LCB counts. Conversely, the partitioned version is only aware of the LCB breakpoints within a given partition, so when merging the intervals across all partitions with high LCB counts, the core genome can degrade in size (Table 2). Better understanding of this phenomenon will require additional experiments on a wider variety of microbial datasets.

Parsnp (p=50) objectively outperformed all other partition sizes, including the effectively nonpartitioned size p=n, on all simulated and real datasets, with the exception of the *M.tuberculosis* dataset. As a result, we have updated Parsnp to default to p=50 when there are over 100 input genomes.

### 3.4 Comparison of resulting phylogenies

As the core-genome size varies across different partitioning sizes, we compared the resulting phylogenies of each partition size for each dataset using the normalized weighted Robinson-Foulds distance (Supplementary Table S1). Except for the *S.aureus* runs, all distances were <0.10, signifying high levels of similarity between the phylogenies. In the *S.aureus* runs, the N-wRF distances were only slightly larger than 0.10, likely due to the partitioned core-genome alignments being twice as large as the nonpartitioned run and slightly more divergent. Altogether, these results show a largely consistent phylogeny across different partition sizes and nonpartitioned runs.

### 3.5 Comparison to Parsnp v1

The experiments in Table 2 include a nonpartitioned run (i.e. p=n) which is algorithmically nearly identical to version 1 of Parsnp. Nonetheless, the software has evolved and so a small direct comparison to Parsnp v1 *vis-a-vis* resource usage is presented here.

We obtained the set of 27 *P. tritici-repentis* genomes (Moolhuijzen *et al.* 2022) used to benchmark Parsnp v1 in a recent study (Fruzangohar *et al.* 2023) and ran Parsnp v2 without partitioning. While most the metrics of Parsnp v2 remain the same as those reported in Fruzangohar *et al.* (2023), the memory usage dropped from 57 to 8.4 Gb.

## 4 Conclusion

Despite approaching the tenth anniversary of its release, Parsnp has an active user-base and remains a widely used method for core-genome alignment of large sets of microbial genomes. Parsnp v2 represents the collective improvements from nearly five years of maintenance and development resulting in several new features, bug-fixes, and enhancements to the user experience. In addition, Parsnp v2 includes a new partitioning strategy which leads to substantial reductions in runtime and memory usage. Collectively, the improvements to Parsnp v2 enable alignments of larger datasets in a package that is more efficient, flexible, robust, and convenient than the original version.

## Supplementary Material

btae311_Supplementary_Data
