# Supplementary material for: Parsnp 2.0: scalable core-genome alignment for massive microbial datasets
Source: Bioinformatics. 2024 May 9;40(5):btae311. doi: 10.1093/bioinformatics/btae311 (PMC11128092; doi:10.1093/bioinformatics/btae311)
Supplement: btae311_Supplementary_Data [file btae311_supplementary_data.pdf]

Parsnp 2.0: Scalable Core-Genome Alignment for Massive Microbial Datasets

April 12, 2024

Contents

1 Supplementary Materials **1**

1.1 Identifying the final core-genome coordinates from a set of partitioned alignments 1

1.2 Merging partitioned alignments 1

1.3 Comparison of phylogenies under different partition sizes 2

1.4 The Parsnp Workflow 3

1 Supplementary Materials

1.1 Identifying the final core-genome coordinates from a set of partitioned alignments

In order to identify the coordinates of the final core genome with respect to a reference sequence, we compute the intersection of the aligned reference regions for each partition. Let q be the number of partitions of n query genomes resulting in p genomes per partition, and $R_i = \{(b_1, e_1), (b_2, e_2), ..., (b_m, e_m)\}$ be the begin and end coordinates of the reference for each of the m LCBs in partition i.

We compute the interval intersection of all R_i interval sets to obtain the coordinates of the final core-genome intervals, R^*. Intervals which are smaller than 10 base pairs are removed from R^*. The output alignments of each partition are then trimmed to match the coordinates in R^*. At the end of this step, each partition will have an alignment file with $|R^*|$ LCBs, where each LCB corresponds to one of the intervals in R^*.

1.2 Merging partitioned alignments

As the merging process is identical across each LCB, we will describe the case for merging a set of LCBs corresponding to the same reference interval. We use two operations, appending and stacking, where appending

LCB_b to LCB_a is the process of adding a each column in LCB_b to the end of LCB_a, and stacking LCB_b on LCB_a is the process of adding the rows in LCB_b to LCB_a. We denote LCB_i as the alignment in partition i. Our goal is merge all LCB_i over all $i \in \{1, 2, ..., q\}$.

In the case of LCBs where there are no indels in the reference for every LCB, the task is straightforward: stack all of the LCBs together. However, special care must be taken when indels are present in the reference sequences. Consider column j of LCB_i, denoted as $LCB_i[j] = [c_1, c_2, c_3, ...c_{1+p}]$, where $LCB_i[j][k] = c_k$ represents the character in row k, column j of LCB_i, and row 0 always corresponds to the reference. If $LCB_i[j][0] = “-”$ and $LCB_i[j][k] \neq “-”$, then we have no information on how the base at $LCB_i[j][k]$ is related to the query sequences from other partitions.

To remedy this, we assemble the output LCB, LCB^* column by column. At column j^* in LCB^*, we keep track of the corresponding column in each of the partition LCBs, denoted by j_i, $i \in q$. If $LCB_i[j_i][k] \neq “-”$ and $LCB_i[j_i][0] = “-”$ for any $i \in [q]$, $k \in [p]$, then instead of stacking all columns and then appending to LCB^*, we append the stacked columns to a temporary LCB X. Once we come across a column where no LCB has any indels in the reference, we compute a multiple sequence alignment A of the sequences in X using SPOA [1], where each sequence is the concatenation of non-gap characters in each row. The alignment A is then appended to LCB^*, X is reset to the empty LCB and the process continues.

1.3 Comparison of phylogenies under different partition sizes

To ensure that downstream results would be similar across different partition sizes, we compared the phylogenies obtained from RAxML [2] using the core variants output by the different Parsnp runs for the bacterial datasets (Table 1). For all three datasets, there were many nearly-identical sequences included which yielded a large number of effectively zero-length branches. To account for this, we used the normalized weighted Robinson-Foulds distance (N-wRF) [3], a branch-length-weighted metric, to compare phylogenies. This is equivalent to the normalized $\mathcal{L}_1$ distance on the branch lengths, where non-shared branches are treated as having length 0 in the other tree. The wRF distance was normalized by the sum of branch lengths across both trees for each comparison.

	M. tuberculosis	*S. aureus*	*K. pneumoniae**
$p = 50$	0.094	0.120	0.068
$p = 100$	0.074	0.119	0.059
$p = 250$	0.043	0.108	–

Table 1: **Normalized weighted Robinson Foulds distance between partitioned phylogenies to the non-partitioned phylogeny.** Parsnp was executed with different partition sizes p on each set of genomes. The resulting phylogenies from each run were compared to the non-partitioned phylogenies using the normalized weighted Robinson-Foulds distance (N-wRF) calculated using DendroPy [4]. *As the non-partitioned version of Parsnp failed to run on the *K. pneumoniae* dataset, we compared the resulting trees to the tree obtained from the $p = 250$.

1.4 The Parsnp Workflow

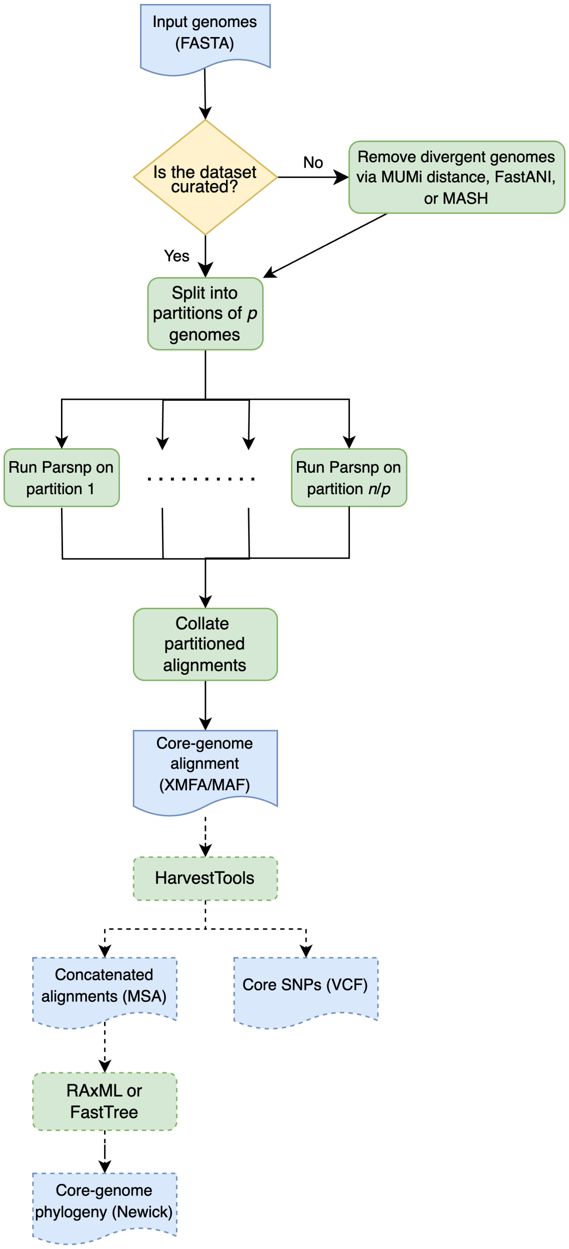

Figure 1: **The Parsnp Workflow.** Steps outlined with dotted lines are optional. `XMFA` is the genome-alignment format used by Parsnp and Mauve [5]. `MAF` is another common genome-alignment format often used by non-core multiple-genome alignment methods.

References

[1] Robert Vaser, Ivan Sović, Niranjan Nagarajan, and Mile Šikić. Fast and accurate de novo genome assembly from long uncorrected reads. *Genome research*, 27(5):737–746, 2017.

[2] Alexandros Stamatakis. Raxml version 8: a tool for phylogenetic analysis and post-analysis of large phylogenies. *Bioinformatics*, 30(9):1312–1313, 2014.

[3] David F Robinson and Leslie R Foulds. Comparison of weighted labelled trees. In *Combinatorial Mathematics VI: Proceedings of the Sixth Australian Conference on Combinatorial Mathematics, Armidale, Australia, August 1978*, pages 119–126. Springer, 2006.

[4] Jeet Sukumaran and Mark T Holder. Dendropy: a python library for phylogenetic computing. *Bioinformatics*, 26(12):1569–1571, 2010.

[5] Aaron CE Darling, Bob Mau, Frederick R Blattner, and Nicole T Perna. Mauve: multiple alignment of conserved genomic sequence with rearrangements. *Genome research*, 14(7):1394–1403, 2004.
